# Pre- and post-natal macronutrient supplementation for HIV–positive women in Tanzania: Effects on infant birth weight and HIV transmission

**DOI:** 10.1371/journal.pone.0201038

**Published:** 2018-10-11

**Authors:** Albert Magohe, Todd Mackenzie, Josephine Kimario, Zohra Lukmanji, Kristy Hendricks, John Koethe, Nyasule Majura Neke, Susan Tvaroha, Ruth Connor, Richard Waddell, Isaac Maro, Mecky Matee, Kisali Pallangyo, Muhammad Bakari, C. Fordham von Reyn, DarDar-2 Study Team

**Affiliations:** 1 Muhimbili University of Health and Allied Sciences, Dar es Salaam, Tanzania; 2 Geisel School of Medicine at Dartmouth and Dartmouth-Hitchcock Medical Center, Lebanon, NH, United States of America; 3 Vanderbilt University School of Medicine, Nashville, TN, United States of America; 4 Tokyo Medical and Dental University, Tokyo, Japan; University of Ghana, GHANA

## Abstract

**Objective:**

To determine if a protein-calorie supplement (PCS) plus a micronutrient supplement (MNS) improves outcomes for HIV-infected lactating women and their infants.

**Design:**

Randomized, controlled trial.

**Setting:**

Dar es Salaam, Tanzania

**Subjects, participants:**

Pregnant HIV-infected women enrolled in PMTCT programs who intended to breastfeed for 6 months.

**Intervention:**

Randomization 1:1 to administration of a PCS plus MNS versus MNS alone among 96 eligible women beginning in the third trimester and continuing for 6 months of breast-feeding.

**Main outcome measure(s):**

Primary: infant weight at 3 months. Secondary: maternal BMI at 6 months.

**Results:**

PCS resulted in significant increases in daily energy intake compared to MNS at all time points (range of differences: +388–719 Kcal); and increases in daily protein intake (range of differences: +22–33 gm). Infant birth weight (excluding twins) was higher in the PCS than MNS groups: 3.30 kg vs 3.04 kg (p = 0.04). Infant weight at 3 months did not differ between PCS and MNS groups: 5.63 kg vs 5.99 kg (p = 0.07). Maternal BMI at 6 months did not differ between PCS and MNS groups: 24.3 vs 23.8 kg/m^2^ (p = 0.68). HIV transmission occurred in 0 infants in the PCS group vs 4 in the MNS group (p = 0.03).

**Conclusions:**

In comparison to MNS the PCS + MNS intervention was well tolerated, increased maternal energy and protein intake, and increased infant birth weight, but not weight at 3 months or maternal BMI at 6 months. Reduced infant HIV transmission in the PCS + MNS group was observed.

**Trial registration:**

Clinical Trials.Gov NCT01461863.

## Background

An estimated 24.7 million people living with HIV reside in sub-Saharan Africa representing 70% of the global HIV burden, and over 90% of HIV-infected women reside in Africa [1]. Statistics for the United Republic of Tanzania report 690,000 women age 15 years and older with HIV infection, and a maternal to child transmission (MTCT) rate of 26% [2].

The interaction between nutrition and HIV infection is complex. Malnutrition and HIV, independently and synergistically, weaken the immune system leading to increased susceptibility to infection and poorer health outcomes [3, 4]. Pregnant and lactating HIV-infected women and their infants living in resource poor settings may be at risk for poorer HIV outcomes due to underlying food insecurity and increased nutrient needs during pregnancy and lactation [5, 6] Dietary deficiencies of both micro- and macro-nutrients have been described in these groups and have led to trials of both types of dietary supplements [3, 7–9].

Trials of micronutrient supplements in HIV-positive pregnant women have shown improvement in maternal and infant health outcomes [10, 11]. However, few randomized controlled trials have evaluated macronutrient supplementation in HIV-infected lactating women.

Nearly two-thirds of HIV-infected pregnant women in Dar es Salaam, Tanzania are energy deficient and nearly half are protein deficient [12], and in a recent pilot study we found that lactating HIV-infected women were also deficient in energy and protein intake [13]. We used these data to design a culturally acceptable protein-calorie supplement for a randomized-controlled trial to assess the impact of macronutrient supplementation on the health outcomes of HIV-infected breast-feeding mothers and their infants.

## Methods

### Study design

The study was designed as a randomized controlled trial to determine if a protein calorie macronutrient supplement (PCS) plus micronutrient supplement (MNS) improved health outcomes in HIV-infected lactating women and their infants compared to MNS alone.

### Subjects

HIV-infected women ≥18 years old in their last trimester of pregnancy were screened from April 2011 to July 2012 at 5 antenatal clinics (3 district hospitals, 2 local clinics) in Dar es Salaam, Tanzania during their last trimester. Eligibility required that the woman be free of acute illness, have a BMI ≥18.5 kg/m^2^ and intend to exclusively breastfeed her infant for at least 3 months. All women were required to be enrolled in anti-retroviral therapy (ART) programs for Prevention of Mother to Child Transmission of HIV infection (PMCT).

### Baseline evaluation

After informed consent, subjects in the third trimester were interviewed to obtain socio-demographic information including age, residence, financial and employment status of both the patient and her partner, highest level of education, income spent specifically on food, and number and ages of persons living in the same household. Phlebotomy was performed for CD4 count and albumin and all subjects had anthropometric evaluations, twenty-four hour dietary recall and food insecurity evaluations.

### Anthropometric measurements

Weight (kg) and, height (recumbent length in infants) were determined following standard protocols and using calibrated instruments. Skin fold thicknesses (mm) at five different areas including the triceps, subscapular, suprailiac, abdomen, and thigh as well as circumferences (cm) around the mid-upper arm, waist, hip, and thigh were determined by one of 3 three trained observers using calibrated Lange skinfold calipers and a Gulick II, non-stretch, pliable tape measure [14].

### Dietary recall

Trained research nutritionists or study nurses evaluated dietary intake using a multiple pass 24-hour dietary recall to list all beverages and foods consumed in the previous day [15]. Multiple passes by the interviewer probed for food preparation and missed food items; standardized food models were used to determine portion size. Energy and protein intakes were then calculated using the Tanzania Food Composition tables [16]. Nutritional education and counseling based on distinct World Health Organization (WHO) and Tanzanian Ministry of Health National Guidelines for HIV-positive women, was provided [17].

### Food insecurity

All subjects were administered the Household Food Insecurity Access Scale (HFIAS), which was adapted from the Food and Nutritional Technical Assistance II Project (FANTA-2) to determine availability and accessibility of food in each woman’s household [18]. An adapted questionnaire contained a total of nine yes or no questions followed by a “frequency-of-occurrence” question. Some questions inquired about the subject’s perception of food vulnerability while other questions addressed the subject’s behavioral responses due to food insecurity. Based on the answers given by the client, a score was assigned to each woman ranging from 0 (no insecurity) to 27 (maximum insecurity).

### Laboratory studies

Phlebotomy was performed to confirm HIV infection and determine the CD4 count. Subjects were required to have two positive rapid HIV tests: Determine HIV-1/2 Ag/Ab (Alere, Chiba, Japan) and Uni Gold HIV-1/2 (Trinity Biotech, Bray, Ireland). Women who tested positive and were unaware of their current HIV status were provided ELISA results, CD4 results, appropriate counseling, referred to a Ministry of Health Care and Treatment Center (CTC) if not already under care and then entered in the study. Subjects with a discordant ELISA result (one positive, one negative) were not eligible and referred to a CTC for further testing.

### Nutritional supplements

In the pilot study the daily dietary intakes of HIV-infected lactating subjects were compared to recommendations of the World Health Organization (WHO) to achieve a mean energy target of 2854 Kcal (which includes an additional 10% increase in energy for HIV infection) and protein target of 86 gm (based on 12% of calories) [13]. Subjects were found to have a median daily dietary energy deficit 2 weeks after delivery of 517 Kcal and a mean daily dietary protein deficit of 29 gm; mean deficits at 6 weeks were 686 Kcal and 36 gm [19–23]. Subjects in the pilot study then participated in focus groups to test the palatability and acceptability several potential protein-calorie supplements (PCS). The supplement that was chosen for the present study was a porridge (Dar-uji) manufactured from locally available ingredients including flour (maize, soybeans, sorghum and millet), full cream dried milk, and sugar (Jamahedo Health Foods, Arusha, Tanzania). The PCS was provided in daily packets containing 1062 Kcal and 42 gm protein, amounts that would cover the 75^th^ percentile of deficits observed in the pilot study. Thirty packets of PCS were given to participants every four weeks. Each 250 mg packet was to be mixed with boiling water and consumed over the course of one day. The micronutrient supplement (MNS) was the standard combination of vitamins and minerals provide to patients with HIV infection by the Ministry of Health of Tanzania (Tishcon Corporation, Salsbury, MD). MNS consisted of 1.4mg thiamine, 1.4 mg riboflavin, 18 mg niacin, 1.9 mg vitamin B-6, 2.6 mg vitamin B-12, 70 ug vitamin C, 10 mg vitamin E and 0.4 mg folic acid [24]. All mothers also received 60 mg ferrous sulfate orally (PO) once daily and 1 mg folic acid PO once daily during pregnancy

### Randomization and intervention

A randomization list was generated by the a study statistician and provided to the study team. Consecutive eligible subjects were randomized 1:1 in block sizes of 4 and 6 to receive the intervention (PCS plus MNS) or control (MNS alone). Subjects were provided with a four week supply. In addition subjects in the PCS intervention group were also given a supply of PCS packets so that 280 Kcal/day was provided for every child less than age 7 years living in the household with the subject.

### Maternal follow-up

All mothers were seen monthly after enrollment in the third trimester at the study clinic, then at home 2–4 weeks after birth, and again at the study clinic monthly for 6 months after infant birth and again at 9 and 15 months after infant birth. At each visit thru 6 months subjects were provided with the supplements, had anthropometric evaluation, and a 24-hour dietary recall. ART use and clinical history were recorded. Laboratory tests including CD4 count were obtained and breast milk samples collected 2–4 weeks after delivery and again 3 months after delivery. Breast milk analysis will be the subject of a separate report.

### Infant follow-up

All infants were examined at the initial home visit and then seen monthly for 6 months, again at 9 months and at 15 months for a medical history and measurement of weight and length. ART use and clinical history were recorded. At 15 months phlebotomy was performed per protocol to perform a single rapid HIV test, Uni Gold HIV-1/2 (Trinity Biotech, Bray, Ireland). Results of HIV testing that had been performed outside the study at government clinics were recorded, typically on infants who had been ill or died. or on infants who had been found to be HIV positive per protocol at 15 months. This testing included results of dried blood spot (DBS) HIV PCR testing at Tanzania Ministry of Health (MOH) HIV clinics where such testing is recommended at 2–3 months for all infants born to HIV-positive mothers. Hospital records were requested on infants who died as inpatients during the study.

### Ethical approval

The study was approved by the Institutional Review Board at Geisel School of Medicine and the Research Ethics Committee of the Muhimbili University of Health and Allied Sciences (MUHAS).

### Sample size, endpoints, and statistical analysis

The primary endpoint for the trial was infant weight at 3 months. A sample size of 96 subjects, 48 subjects in each group, was calculated based on type 1 and 2 error rates of 5% and 20% respectively, a minimally clinically effect size of 0.5 kg, assuming that the standard deviation of weight at 2 months among HIV infected infants born on the African continent is approximately 0.7 kg (IQR = 4.0 to 4.9 kg. [25].

The primary endpoint, infant weight at 3 months, was compared between the two study arms using a two-sample t-test.

The secondary endpoint was maternal BMI 6 months after delivery. Maternal BMI (as well as weight and mid upper arm circumference) at 6 months post-delivery was compared between the two study arms using a linear model that controlled for maternal BMI at baseline. Laboratory parameters including maternal CD4 were compared in the same manner. Dietary intakes were compared between the two arms at specific time points using t-tests. Infant mortality was compared between the two study arms using Fisher’s exact test. The incidence of infant HIV infection as determined by DBS at 2 months or HIV ELISA at 15 months was compared between the study arms using Fisher’s exact test and also a log rank test that censored follow-up at 18 months or death. The main analyses included all mothers and infants. As a sensitivity analysis we repeated analyses excluding mothers of twins, and twins.

## Results

### Patient characteristics

**Fig 1:**
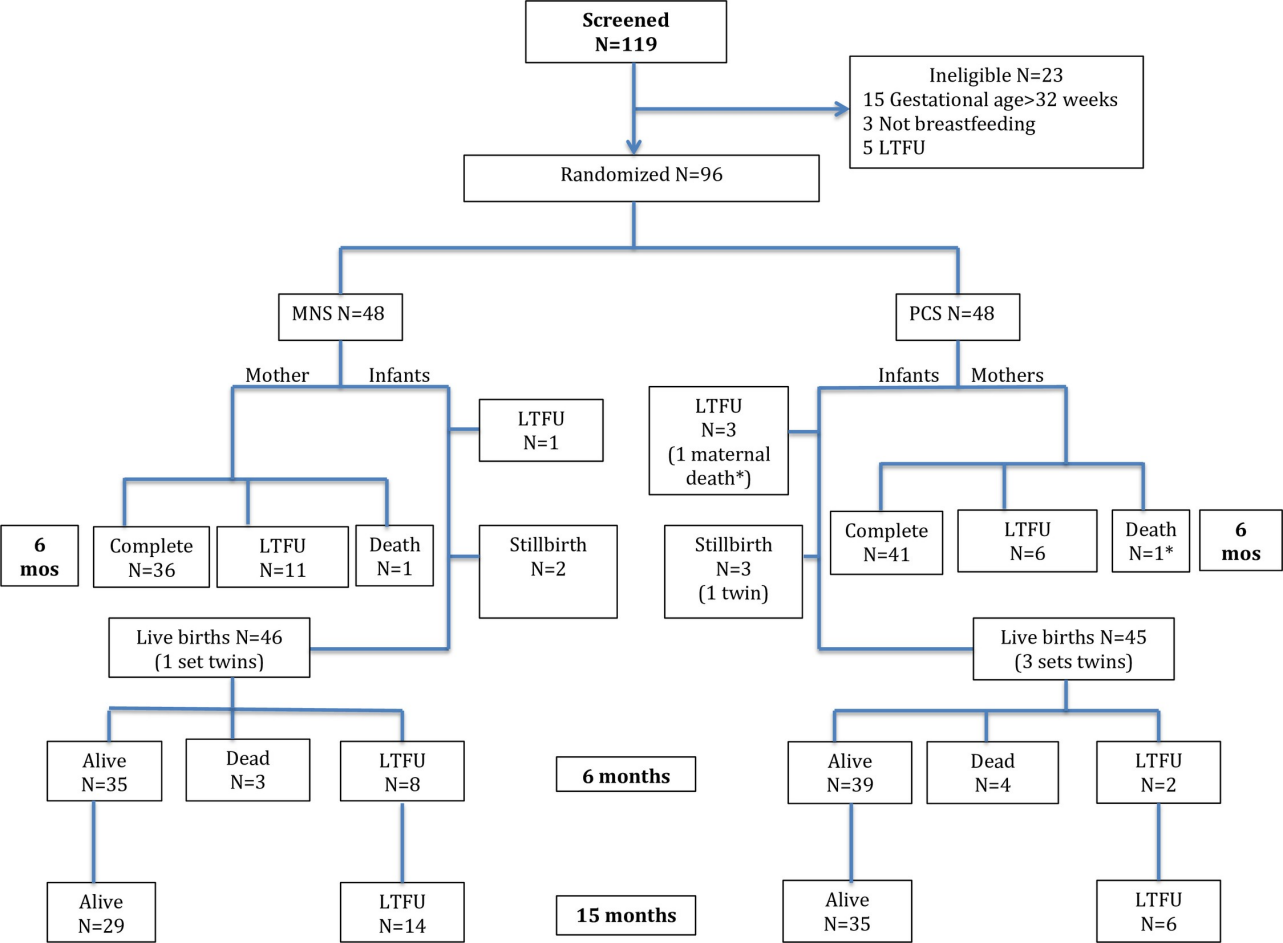
Consort diagram.

A total of 119 subjects were screened and 96 enrolled in the study during their third trimester between April 21, 2011–July 9, 2012 (Fig 1). Subjects were randomized 1:1 to the 2 intervention groups. Baseline characteristics, including anthropometry and food insecurity, did not differ between the two groups (Table 1).

**Table 1:** Baseline subject characteristics in third trimester.

	MNS	PCS +MNS
Mean age, years	31	31
Education, N (%)		
None	3 (7%)	6 (13%)
Some primary	5 (11)	1 (2)
Primary	26 (57)	30 (63)
Secondary	3 (7)	3 (6)
Higher than secondary	9 (20)	8 (17)
Marital status, N (%)		
Single	8 (17%)	8 (17%)
Married	32 (67)	32 (67)
Divorced	2 (4)	2 (4)
Widowed	0 (0)	1 (2)
Cohabitating	5 (10)	5 (10)
Employed, N (%)	22 (47%)	27 (56%)
Partner employed, N (%)	40 (93%)	38 (93%)
Mean number of people in household,	5.1	2.8
Mean Tanzanian shillings spent on food per household/day *(USD)*	6287 TSh ($2.99)	5341 TSh ($2.54)
Daily activity level, N (%)		
Very Light	0	2
Light	32	26
Moderate	14	19
Heavy	0	0
Food Insecurity Score	6.6	5.0
Weight *(mean*, *kilograms)*	63.8	62.4
BMI *(mean*, *kg/m*^*2*^*)*	25.8	25.3
Mid-upper arm circumference *(mean*, *cm)*	27.2	27.2
Hematocrit (mean)	29.1	28.8
Albumin (mean)	3.32	3.34
CD4 count (mean/mm^3^)	394	469
N on Antiretroviral therapy (%)	38 (79%)	37 (77%)

At baseline in the third trimester a total of 38/48 (79%) women in the MNS group and 37/48 (77%) women in the PCS group were on ART (p = 1.0) A total of 9/47 (19%) of women in the MNS group and 7/46 (15%) in the PCS group reported occasionally missing a day or more of ART during the third trimester (p = 0.82). A total of 19/29 (66%) women in the MNS group and 17/35 (49%) women in the PCS group reported remaining on ART at 3 months (p = 0.27). A total of 14/28 (50%) women in the MNS group and 18/34 (53%) women in the PCS group reported remaining on ART at 6 months (p = 1.0). At the time of delivery 44/48 (92%) infants in the MNS group and 42/48 (88%) infants in the PCS were on ART (p = 0. 74).

### Dietary recall

Baseline dietary intakes were comparable in the two intervention groups (Table 2). At the 2–4 week post-delivery home visit women in the PCS group had a daily energy intake 415 Kcal higher than the MNS group and a daily protein intake 22 gm higher than the MNS group. Compared to WHO recommendations these 2–4 week values represent a deficit of 81 Kcal and 6 gm protein in the PCS group and a deficit of 496 Kcal and 28 gm protein in the MNS group. Differences in dietary energy and protein intake between the two groups persisted through 6 months post-partum. Diarrhea, nausea, or vomiting were not reported by women in either group. Death or loss to follow-up at 6 months was 25% (12/48) in the MNS group and 15% (7/48) in the PCS group (p = 0.31). Information on causes of death in mothers and infants was not available.

**Table 2:** Mean (sd) energy and protein intake of study subjects.

	MNS	PCS + MNS	p-value
Baseline*			
**Kcal**	2185 (721)	2312 (683)	0.39
**Protein**	55 (30)	58 (23)	0.62
Pre-birth**			
**Kcal**	2161 (658)	2880 (591)	<0.001
**Protein**	52 (26)	85 (231)	<0.001
**Week 2–4 post delivery**			
**Kcal**	2358 (677)	2773 (689)	0.007
**Protein**	58 (20)	80 (27)	<0.001
**Month 3 post delivery**			
**Kcal**	2462 (612)	2850 (712)	0.03
**Protein**	60 (27)	80 (26)	0.004
**Month 6 post delivery**			
**Kcal**	2257 (616)	2896 (460)	<0.001
**Protein**	51 (16)	82 (17)	<0.001

* Data not available for 3 subjects at baseline and additional subjects during follow-up

** Includes subjects who had been taking the supplement for at least one month

Note: Data and statistical significance similar when excluding women with twins

### Study endpoints and anthropometry

Primary and secondary endpoints are shown in Table 3. Infant weights at 3 months did not differ between the two groups (primary endpoint). Among 52 women on ART at 3 months mean infant weight at 3 months was 5.41 in the MNS group and 5.81 kg in the PCS group (p = 0.08). Among 36 women not on ART at 3 months mean infant weight at 3 months was 6.0 kg in the MNS group and 5.6 kg in the PCS group (p = 0.22).

**Table 3:** Infant weights and maternal BMI values.

	MNS	PCS+MNS	P-value	Mean difference (sem)
**Infant weight (kg, mean, sd)**				
Birth, all infants	3.01 (0.61)	3.17 (0.59)	0.21	0.16(0.13)
Birth, no twins	3.04 (0.60)	3.30 (0.57)	0.04	0.26 (0.13)
Month 3, all infants*	5.99 (0.73)	5.63 (0.89)	0.07	-0.36 (0.20)
Month 3, no twins	6.05 (0.63)	5.77 (0.83)	0.14	-0.28 (0.19)
Month 6, all infants	7.32 (1.32)	6.92 (0.82)	0.13	-0.40 (0.22)
Month 6, no twins	7.42 (1.25)	6.99 (0.84)	0.12	-0.43 (0.23)
**Infant z score (weight for length)**				
Month 3, all infants	0.23 (1.18)	0.24 (1.31)	0.96	0.01 (0.33)
Month 3, no twins	0.27 (1.11)	0.30 (1.34)	0.90	0.03 (0.31)
Month 6, all infants	0.15 (1.53)	-0.14 (1.08)	0.07	-0.29 (0.35)
Month 6, no twins	0.15 (1.51)	-0.10 (1.12)	0.07	-0.25 (0.43)
**Maternal BMI (kg/m^2^, median)**				
Baseline^	25.8	25.3	0.58	-0.5 (0.9)
Post-delivery	24.3	23.5	0.34	-0.8 (0.9)
Month 3	25.2	24.9	0.73	-0.3 (1.0)
Month 6**	24.3	23.8	0.68	-0.5 (1.1)
**Maternal MUAC (cm, mean)**				
Pre-birth	27.2	27.2	0.97	-0.0 (0.8)
Month 3	27.8	27.3	0.56	-0.5 (0.9)
Month 6**	28.0	27.9	0.88	-0.1 (1.1)
**Maternal CD4 increase (cells/mm^3^)**				
Month 6	135	161	0.64	26 (41)

* Primary study endpoint

** Secondary study endpoint

MUAC = mid-upper arm circumference

Maternal BMI at 6 months did not differ between the two groups (secondary endpoint). Change in maternal BMI at 6 months did not differ between the two groups (data not shown). Trends in BMI differences at 6 months differed by ART status: for 38 mothers on ART median BMI was 25 in the MNS group and 22 in the PCS group (p = 0.07); for 39 mothers not on ART median BMI was 22 in the MNS group and 24 in the PCS group (p = 0.23). MUAC values were not significantly different at baseline or at 3 and 6 months. Additional anthropometric data will be the subject of a separate report on body composition.

### Laboratory studies

There were no significant differences in CD4, albumin, hemoglobin or white blood count between the 2 groups at baseline or during follow up and no significant difference in change in of these laboratory values from baseline to 3 or 6 months between the two groups (data not shown). For CD4 count the mean increases at 6 months were 135/mm^3^ in the MNS group and 161/mm^3^ in the PCS group (p = 0.64) and for hematocrit 4.4 and 2.3 respectively (p = 0.16).

### Infant HIV infection and mortality

As shown in Table 4 the rate of infant HIV infection by age 15 months was 8.3% (4 of 48) in MNS group and 0% (0 of 48) in PCS group (p = 0.117, Fisher’s exact test; p = 0.036, log-rank test). The 4 cases of HIV transmission included 2 cases confirmed by ELISA (one tested at 15 months per-protocol and one tested at 8 months during a terminal hospitalization) and 2 cases confirmed by Ministry of Health dried blood spot (DBS) PCR testing at 2–3 months. There were 7 infant deaths in each treatment group (p = 0.90)

**Table 4:** Infant HIV infection.

Study number	Group	Baseline Maternal CD4	Antiretroviral therapy				Infant feeding	Positive HIV test	Infant outcome
			Maternal delivery	Infant birth	Maternal 3 mos	Infant 3 mos			
**4303**	MNS	121	AZT NVP	NVP	None	AZT 3TC NVP	Mixed	PCR 2 mos	Died 8 mos
**4309**	MNS	624	None	AZT NVP	None	AZT 3TC NVP	Breast	ELIS 15 mos	Alive 15 mos
**4383**	MNS	389	AZT	NVP	None	None	Breast	ELIS 8 mos	Died 10 mos
**4388**	MNS	60	AZT 3TC NVP	NVP	AZT 3TC NVP	AZT 3TC NVP	Breast	PCR 3 mos ELISA5 mos	Alive 15 mos

AZT = azidothymidine; ELISA = enzyme linked immunosorbent assay for HIV; Mixed = breast milk plus formula: NVP = nevirapine; PCR = dried blood spot polymerase chain reaction (PCR) for HIV

## Discussion

In the present study we found that a locally produced protein-calorie supplement was well-accepted and well-tolerated by HIV-infected lactating women in Tanzania who were not malnourished at baseline. The PCS led to a measurable increase in dietary energy and protein intake and erased the calculated baseline deficits compared to recommendations of the WHO. However, the PCS did not affect the primary endpoint of infant weight at 3 months or the secondary endpoint of maternal BMI at 6 months. However, HIV transmission was unexpectedly lower among infants of mothers who received the supplement.

Although not a primary study endpoint, birth weight was higher in the PCS group and significantly higher when twins were excluded from the analysis. Lower infant birth weight has been consistently associated with poorer health outcomes in children [26]. Higher birth weight in the intervention group may have been a result of the PCS providing higher energy and protein intake during the last trimester of pregnancy. The absence of an effect on infant weight at 3 months is consistent with maintenance of lactational capacity and thus protection of infant growth, in women not severely malnourished [27]. The absence of an effect on infant weight at 3 months could also be due to cultural practices ensuring that lactating women receive preferential access to food, though 24 hour recalls continued to show inadequate intakes in the MNS group.

Since all women in the trial were required to be enrolled in PMCT programs the trial was not powered to detect an effect of the PCS supplement on rates of mother-to-infant HIV transmission. However, HIV transmission rates were significantly lower in the PCS group despite similar, though low, overall rates of maternal ART at 3 and 6 months. Since HIV testing data and complete follow-up was not available on all infants we cannot exclude the possibility that this was a chance finding. However, mothers in the trial faithfully reported infant illness and outside HIV test results so we believe unreported infant HIV infections were unlikely. Maternal nutrition could have affected HIV transmission risk in several ways: through improved ART compliance, reduced ART side effects, reduction in mixed infant feeding, or improved breast milk characteristics. Limited data from this study do not support differences in ART compliance or side effects or differences in rates of mixed feeding.

The present study adds to available data on macronutrient supplementation for HIV-infected lactating women. In Malawi a lipid-based macronutrient supplement for HIV-infected lactating women had no effect on infant growth[28]. In another trial in Malawi, the Breastfeeding, Antiretrovirals and Nutrition (BAN) study HIV-infected mothers and their infants were randomly assigned to either receive a lipid based nutrient supplement (LNS) providing 700 calories and 20 grams of protein daily, or control. While the LNS reduced weight loss among HIV-infected breastfeeding women the supplement had no effect on infant growth [29, 30]. In the present study macronutrient supplementation did not have an effect on infant weight or maternal BMI.

This study has several limitations. The relatively small sample size could have prevented detection of a modest treatment effect. In addition it would not have been ethical to enroll and randomize women with baseline malnutrition, a group that would have been more likely to benefit from the intervention. The reported absence of nausea, vomiting or diarrhea in study subjects over 6 months is unexpected and may indicate a reluctance to admit these symptoms. Finally, without serial PCR testing on all infants we are unable to determine the timing of infant HIV infection. Breast milk transmission would have been possible for some of the 4 infections since 3 of the mothers were no longer on ART at 3 months.

In summary we found that a locally produced protein-energy supplement administered for 6 months to non-malnourished lactating women with inadequate pre-study dietary intake did not have an effect on infant weight at 3 months or maternal BMI at 6 months. An unexpected decrease in the rate of HIV infection among infants of mothers receiving the supplement remains unexplained.

## Supporting information

S1 File CONSORT 2010 Checklist. (DOC)

S2 File Protocol. (PDF)

S3 File (ZIP)

## References

[1] UNAIDS. 2012 Regional Fact Sheet. 2012.

[2] UNAIDS. United Republic of Tanzania, Country progress reporting March 2012. 2012.

[3] Koethe JR, Chi BH, Megazzini KM, Heimburger DC, Stringer JS. Macronutrient supplementation for malnourished HIV-infected adults: a review of the evidence in resource-adequate and resource-constrained settings. Clin Infect Dis. 2009;49(5):787–98. Epub 2009/07/25. 10.1086/605285 ; PubMed Central PMCID: PMC3092426. 19624276 PMC3092426

[4] Rawat R, McCoy SI, Kadiyala S. Poor diet quality is associated with low CD4 count and anemia and predicts mortality among antiretroviral therapy-naive HIV-positive adults in Uganda. J Acquir Immune Defic Syndr. 2013;62(2):246–53. Epub 2012/11/03. 10.1097/QAI.0b013e3182797363 . 23117502

[5] Villamor E, Saathoff E, Msamanga G, O'Brien ME, Manji K, Fawzi WW. Wasting during pregnancy increases the risk of mother-to-child HIV-1 transmission. J Acquir Immune Defic Syndr. 2005;38(5):622–6. Epub 2005/03/29. . 15793376 10.1097/01.qai.0000143601.48986.47

[6] Raiten DJ, Mulligan K, Papathakis P, Wanke C. Executive summary—nutritional care of HIV-infected adolescents and adults, including pregnant and lactating women: what do we know, what can we do, and where do we go from here? Am J Clin Nutr. 2011;94(6):1667S–76S. Epub 2011/11/18. 10.3945/ajcn.111.019711 ; PubMed Central PMCID: PMC3226019. 22089438 PMC3226019

[7] Papathakis P, Van Loan M, Rollins N, Chantry C, Bennish M, Brown K. Body composition changes during lactation in HIV-infected and HIV-uninfected South African women. J Acquir Immune Defic Syndr. 2006;43:467–74. 10.1097/01.qai.0000243094.42276.92 16980904

[8] Papathakis PC, Rollins NC, Chantry CJ, Bennish ML, Brown KH. Micronutrient status during lactation in HIV-infected and HIV-uninfected South African women during the first 6 months after delivery. Am J Clin Nutr. 2007;85:182–92. 10.1093/ajcn/85.1.182 17209195

[9] Papathakis PC, Pearson KE. Food fortification improves the intake of all fortified nutrients, but fails to meet the estimated dietary requirements for vitamins A and B6, riboflavin and zinc, in lactating South African women. Public health nutrition. 2012;15(10):1810–7. Epub 2012/08/10. 10.1017/S1368980012003072 . 22874138

[10] Fawzi WW, Msamanga GI, Spiegelman D, Wei R, Kapiga S, Villamor E, et al A randomized trial of multivitamin supplements and HIV disease progression and mortality. N Engl J Med. 2004;351(1):23–32. Epub 2004/07/02. 10.1056/NEJMoa040541 . 15229304

[11] Fawzi WW, Msamanga GI, Urassa W, Hertzmark E, Petraro P, Willett WC, et al Vitamins and perinatal outcomes among HIV-negative women in Tanzania. N Engl J Med. 2007;356(14):1423–31. Epub 2007/04/06. 10.1056/NEJMoa064868 . 17409323

[12] Lukmanji Z, Hertzmark E, Spiegelman D, Fawzi WW. Dietary patterns, nutrient intake, and sociodemographic characteristics in HIV-infected Tanzanian pregnant women. Ecology of food and nutrition. 2013;52(1):34–62. Epub 2013/01/04. 10.1080/03670244.2012.705768 . 23282190

[13] Kim F, Neke NM, Hendricks K, Wamsele J, Lukmanji Z, Waddell R, et al Deficiencies of macronutrient intake among HIV-positive breastfeeding women in Dar es Salaam, Tanzania. J Acquir Immune Defic Syndr. 2014;67(5):569–72. Epub 2014/09/18. 10.1097/QAI.0000000000000352 ; PubMed Central PMCID: PMC4229458. 25230293 PMC4229458

[14] Gibson RS. Principles of Nutritional Assessment. Oxford: Oxford University Press; 2005.

[15] Raper N, Perloff B, Ingwersen L, Steinfeldt L, Jaswinder A. An overview of USDA’s dietary intake data system. J Food Compos Anal 2004;17:545–55.

[16] Lukmanji Z, Hertzmark E, Mlingi N, Assey V, Ndossi G, Fawzi WW. Tanzania food composition tables. Muhmibili University of Health and Allied Sciences, Dar es Salaam, Tanzania—Harvard School of Public Health. 2008.

[17] Papathakis P, Rollins N. HIV and nutrition: pregnant and lactating women Consultation on Nutrition and HIV/AIDS in Africa: Evidence, lessons and recommendations for action: World Health Organization; 2005.

[18] Coates J, Swindale A, Bilinsky P. Food and Nutrition Technical Assistance Project (FANTA): Household Food Insecurity Access Scale (HFIAS) for Measurement of Food Access: Indicator Guide, Version 2.2006.

[19] Kim F, Neke NM, Hendricks K, Wamsele J, Lukmanji Z, Waddell R, et al Brief Report: Deficiencies of Macronutrient Intake Among HIV-Positive Breastfeeding Women in Dar es Salaam, Tanzania. J Acquir Immune Defic Syndr. 2014;67(5):569–72. Epub 2014/09/18. 10.1097/QAI.0000000000000352 ; PubMed Central PMCID: PMC4229458. 25230293 PMC4229458

[20] WHO, editor Human energy requirements. Joint FAO/WHO/UNU Expert Consultation; 2004 17–24 October 2001; Rome, Italy.

[21] WHO. Food and Agriculture Organization of the United Nations/World Food Organization of the United Nations (FAO/WHO) Expert Committee. Energy and Protein Requirement. WHO Technical Report Series, No. 724, Geneva. 1985.

[22] Hsu JWC, editor Macronutrients and HIV/AIDS: a review of current evidence. Consultation on nutrition and HIV/AIDS in Africa: evidence, lessons, and recommendations for rction; 2005; Durban, South Africa.

[23] WHO, editor Executive Summary of a Scientific Review: Consultation on Nutrition and HIV/AIDS in Africa: Evidence, Lessons, and Recommendations for Action. 2005; Durban, South Africa.

[24] Kawai K, Kupka R, Mugusi F, Aboud S, Okuma J, Villamor E, et al A randomized trial to determine the optimal dosage of multivitamin supplements to reduce adverse pregnancy outcomes among HIV-infected women in Tanzania. Am J Clin Nutr. 2010;91(2):391–7. Epub 2009/11/27. 10.3945/ajcn.2009.28483 ; PubMed Central PMCID: PMC2806894. 19939985 PMC2806894

[25] Violari A, Cotton MF, Gibb DM, Babiker AG, Steyn J, Madhi SA, et al Early antiretroviral therapy and mortality among HIV-infected infants. N Engl J Med. 2008;359(21):2233–44. Epub 2008/11/21. 10.1056/NEJMoa0800971 ; PubMed Central PMCID: PMC2950021. 19020325 PMC2950021

[26] MciIntire DD, Bloom SL, Casey BM, Leveno KJ. Birth weight in relation to morbidity and mortality among newborn infants. N Engl J Med. 1999;340:1234–8. 10.1056/NEJM199904223401603 10210706

[27] Lactation CoNSDPa. Nutrition During Pregnancy and Lactation: An Implementation Guide. In: National Academy of Sciences IoM, Food and Nutrition Board, editor.; Washington, DC: National Academy Press; 1992.

[28] Flax VL, Bentley ME, Chasela CS, Kayira D, Hudgens MG, Knight RJ, et al Use of lipid-based nutrient supplements by HIV-infected Malawian women during lactation has no effect on infant growth from 0 to 24 weeks. J Nutr. 2012;142(7):1350–6. Epub 2012/06/01. 10.3945/jn.111.155598 ; PubMed Central PMCID: PMC3374670. 22649265 PMC3374670

[29] Kayira D, Bentley ME, Wiener J, Mkhomawanthu C, King CC, Chitsulo P, et al A lipid-based nutrient supplement mitigates weight loss among HIV-infected women in a factorial randomized trial to prevent mother-to-child transmission during exclusive breastfeeding. Am J Clin Nutr. 2012;95(3):759–65. Epub 2012/01/20. 10.3945/ajcn.111.018812 ; PubMed Central PMCID: PMC3278250. 22258269 PMC3278250

[30] Jamieson DJ, Chasela CS, Hudgens MG, King CC, Kourtis AP, Kayira D, et al Maternal and infant antiretroviral regimens to prevent postnatal HIV-1 transmission: 48-week follow-up of the BAN randomised controlled trial. Lancet. 2012;379(9835):2449–58. Epub 2012/05/01. 10.1016/S0140-6736(12)60321-3 ; PubMed Central PMCID: PMC3661206. 22541418 PMC3661206

