# Supplementary material for: Pre- and post-natal macronutrient supplementation for HIV–positive women in Tanzania: Effects on infant birth weight and HIV transmission
Source: PLoS One. 2018 Oct 11;13(10):e0201038. doi: 10.1371/journal.pone.0201038 (PMC6181269; doi:10.1371/journal.pone.0201038)
Supplement: S2 File — (PDF) [file pone.0201038.s002.pdf]

DarDar 2-B Nutrition Study
Scientific Protocol

1. Summary. The DarDar 2-B Nutrition Study is a randomized, controlled trial of 9 months of a porridge protein-calorie supplement (PCS) and micronutrient supplement (MNS) vs MNS control alone administered to 96 HIV-infected women from Dar es Salaam, Tanzania in their third trimester who plan to exclusively breastfeed for 6 months. The objective is to determine if a PCS will improve health outcomes in these mothers and their infants. The study design is based on an earlier data-gathering study (DarDar 2-A Nutrition Study) which determined dietary intakes, food preferences and the extent of malnutrition in the target group.

Two central issues underscore the rationale for this study: 1) HIV-infected women have increased nutritional demands and/or deficits which are augmented/exacerbated by pregnancy and lactation, and 2) It is highly beneficial for infants in resource-limited settings to breastfeed, even (and especially) in the setting of maternal HIV infection. We hypothesize that optimized nutrition through provision of a culturally acceptable PCS will result in improved maternal and infant health mediated by improved infant growth, improved lactation, better control of maternal viral replication and maintenance of immune function, reduced infant exposure to maternal virus via breast milk, and enhanced maternal-infant transfer of passively acquired immune factors.

2. Recruitment and eligibility. HIV-infected women in their second trimester will be recruited by the DarDar Study Nurse from pre-natal clinics at designated hospitals and dispensaries in Dar es Salaam including, but not limited to, facilities at Mbagala, Temeke, Mwananyamala, and Amana Hospitals to begin the study in their third trimester. The protocol will be explained and signed consent obtained. Eligibility criteria include expected residence in Dar during the duration of breastfeeding, willingness to identify a confidante (friend, spouse or relative), minimum age 18 and plan to exclusively breastfeed. Subjects with defined high risk pregnancy (e.g., diabetes, pre-eclampsia) who are typically seen at speciality clinics at district hospitals will be excluded. Subjects with allergy to components of the PCS will also be excluded.

3. Baseline studies. All subjects will be given a standardized interview consisting of HIV medical history, and a standardized nutritional assessment (see Section 11) based on a 24-hour recall; a body composition evaluation (height, weight, 3-site skinfold thickness, circumference of upper arm, waist and hip); and laboratory studies (serum albumin, and CBC). Phlebotomy will be performed for CD4 count, and breast milk will be obtained for analysis. All subjects will receive nutritional counseling for pregnancy and breastfeeding and counseling on the risks and benefits associated with exclusive breastfeeding.

At the end of the baseline visit, each subject will be asked to complete a brief questionnaire known as the Informed Consent Evaluation and Feedback Tool (ICE-FT); this has been developed to improve comprehension of the informed consent process and measure how well subjects understand what the study involves and the risks/benefits of participation.

4. Randomization and intervention. A master randomization list will be generated by the Data Center at Dartmouth based on 1:1 assignment to PCS/MNS or MNS alone (control). Eligible subjects will be assigned a study number from the randomization list which will determine their assignment. Subjects in the PCS group will receive a supply of the PCS/MNS; subjects in the MNS group will receive a one month supply of MNS. In addition to the MNS women in both groups will be given a thermos for storing prepared foods.

5. MNS and PCS. Composition of the PCS is based on data obtained in Study 2-A. The caloric and protein intakes observed in Study 2-A were compared to recommendations of the World Health Organization (WHO) for pregnant and lactating women with HIV and the respective deficits in kcal/d and gm protein/d calculated. The PCS is formulated to cover the 75th percentile of the average calculated kcal and protein deficits in women from Study 2-A. All subjects will be counseled to continue their regular diets and consume the MNS and/or PCS as an additional supplement.

MNS during pregnancy and lactation. The MNS (micronutrient supplement) will be a tablet consisting of the standard Tanzanian Ministry of Health multivitamin (1 RDA) for pregnant and lactating women.

Iron and folate during pregnancy. Pregnant subjects should also be receiving 60 mg ferrous sulfate PO once daily and 1 mg folic acid PO once daily for the duration of pregnancy. If they are not then iron and folate will be provided each month by the Study. Subjects will be asked to discontinue other vitamin or mineral supplements they may be taking.

Porridge Protein Calorie Supplement (PCS). The PCS, designated "Dar-uji" (uji = Kiswahili for porridge) will be a dry packaged mixture to be cooked with water to prepare the PCS porridge.

Table 1. Dar-uji protein calorie supplement (PCS)

Composition per 250g package	DarDar Lishe flour* 100 g<br>Full cream dried milk 100 g<br>Sugar 50 g<br>TOTAL 250 g
Daily serving	250 gm diluted in 900 mL water, boiled, stored in thermos and divided into 3 between-meal feedings
Calories	1062 kcal per 250 gm (354 kcal per serving x 3)
Protein	41.7 gm per 250 gm [15.6%] (13.9 gm per serving x 3)
Frequency of distribution for study subject	15 packets every 2 weeks
Allowance for each child ≤ age 7 in household	4 packets every 2 weeks (280 kcal/day)

*(maize 50g, soy beans 25g, sorghum flour 12.5g, millet flour 12.5g)

Distribution and monitoring of PCS. PCS subjects will visit the study clinic monthly for a new supply of the PCS and will be trained to record their daily consumption on a monitoring form. The form will be reviewed by the research worker at each visit. Current breastfeeding practices will be recorded at each study visit for all subjects. In addition, random home visits will be conducted to assess compliance and food sharing.

Sharing of PCS. For each child of age ≤ 7 living in the same household as the subject we will provide a child's food supplement of Dar-uji (separate color packaging) equal to approximately 280 kcal/day.

6. Follow-up. Mothers (and infants) will have monthly study visits for the duration of the intervention at the DarDar study clinics at the IDC and reimbursed for travel for each visit. Following the 9 month treatment

intervention, mothers and infants will be evaluated again after 3 months (= infant month 9 below) and again after another 6 months (= infant month 15 below) for a minimum of 15 months after delivery for all mother-infant pairs. At each visit an interview will be conducted to assess intercurrent illness (e.g. infant diarrhea, maternal mastitis), a physical examination conducted, height and weight recorded and a 24 hour recall and standardized 24 hour nutritional recall performed (see Section 11). Additional studies, as outlined in Table 2 (below) will include maternal phlebotomy for CD4 count, CBC and albumin levels, and breast milk sampling. Infants will be tested for HIV by ELISA at 15 months. Infant height and weight will be monitored at each study visit.

Table 2: Summary of Study B protocol beginning with baseline (BL) evaluation

		Intervention: PCS + MNS vs MNS alone											
		3rd Trimester			Delivery and breastfeeding								
**MONTHLY evaluations**	**BL**	**-3**	**-2**	**-1**[a]	**0**	**1**	**2**	**3**	**4**	**5**	**6**	**9**	**15**
**24 hour recall**													
Mother	x	x	x	x	x	x	x	x	x	x	x	x	x
**History/physical**													
Mother[b]	x	x	x	x	x	x	x	x	x	x	x	x	x
Infant					x	x	x	x	x	x	x	x	x
Infant ultrasound	x												
**Laboratory**													
Mother													
CBC/albumin	x				x						x		
CD4 T cells	x				x			x					
Store plasma[e]	x										x		
Breast milk													
• Immune factors[c]					x			x					
• Composition[d]					x			x					
Infant													
HIV ELISA													x[e]

[a] Number of monthly pre-natal visits will vary from 1-3 depending on how early mother is enrolled in third trimester; when possible the baseline visit (BL) will typically be the same as the -3 visit.
[b] A physical examination will be conducted and nutritional assessment performed including weight, height, 3-site skinfold thickness (triceps, subscapular and suprailiac) and upper arm, and waist and hip circumference. During pregnancy measurements will include only, height, weight, mid arm circumference and triceps skinfold thickness. Fat free body mass and percent body fat will be calculated from anthropometric measurements using the equations of Durnin et al. Upper arm circumference and triceps skinfold measures will be used to calculate mid-arm muscle circumference or area.
[c] Pro-inflammatory cytokines IL-6 and IL-8 and sIgA will be measured as described below
[d]Percentage fat content will be measured by standard creamatocrit.
[e]Plasma will be frozen and stored for possible additional assays of nutritional, virologic and/or immunologic status.

7. Sample size. The primary endpoint will be infant weight at 3 months. Our hypothesis is that infants of mothers in the active PCS treatment group will have a median 3 month weight 0.5 kg higher than infants of mothers in the MNS control group. The standard deviation of infant weight at 2 months among HIV infected infants born on the African continent is approximately 0.7 kg (IQR of 4.0 to 4.9kg; Violari NEJM 2008). We plan to enroll 96 women in the third trimester of pregnancy, and randomize 48 to each arm. We anticipate that at least 95% of enrolled mothers will give birth to a live infant who survives to 3 months, and that no more than 5% of these mother-infant pairs will be lost to follow-up. Thus we anticipate obtaining the primary endpoint on 85 infants. This gives us 80% power to detect effects of nutrition on infant weight at 3 months of 0.50 kilograms. (1.1 pounds).

8. Data analysis. For the primary endpoint, median infant weight, will be compared between the two groups at 3 months using intention to treat (ITT) methodology. Multivariate analysis will be used to control for variables which differ between PCS and MNS treatment arms at baseline (e.g. maternal CD4 at baseline, infant birth weight). The secondary endpoint will be maternal BMI at the end of the intervention (6 months after infant birth).

The method of generalized estimating equations will be used to accommodate repeated measurements of infant weight during follow-up. The cluster unit is the subject and the correlation structure will be unstructured. In addition weight at other time points (e.g., birth, 6 months) will be compared between the two arms using a t-test.

We will use two methods to assess the influence of on compliance on endpoints. The first will be an as-treated analysis done using proportion of supplements reported taken as the exposure. We will test if this correlates with infant weight at 3 months using linear regression to adjust for any baseline covariates that are associated with proportion compliance. As-treated analyses are observational analyses that are subject to bias. Thus, the second approach will be to conduct an instrumental variable analysis which has the benefit of avoiding selection bias. Instrumental variable analysis (based on randomization) will yield an unbiased estimate of the effect of compliance on infant weight at 3 months. Subgroup analyses will be conducted on women entering the study in the lowest BMI quartile.

Additional exploratory endpoints will be infant development, infant diarrheal disease, maternal CD4, maternal albumin, breast milk composition, and breast milk immunological parameters.

9. Health Care.

Mothers: Routine medical care including treatment of intercurrent infections (e.g., pneumonia, thrush etc), administration of septrim/bactrim (sulfa-co-trimoxazole) per MOH guidelines, and referral for hospitalization will be provided for all adult study subjects at the adult DarDar IDC site. Anti-retroviral therapy (ART) will be provided thru Ministry of Health HIV Care and Treatment Centers (CTCs) according to current national guidelines for prevention of maternal to child transmission of HIV (PMTCT). We expect all mothers who are not already on ART to have ART instituted before delivery per MOH guidelines. At each visit study staff will ensure that ART-eligible subjects are referred to a CTC facility.

Infants: Routine infant evaluation for HIV infection will be performed by the Ministry of Health (MOH) as part of the standard PMTCT program . It is expected that newborn infants of the HIVpositive mothers in the study will be started on ART if they have a positive PCR for HIV at birth. HIV-positive infants will be enrolled in the DarDar Pediatric Program (DPP) for HIV care and ART treatment. HIV-negative infants will have routine

study visits performed at the adult DarDar IDC site and referred to the DPP for evaluation and treatment of intercurrent health problems. Collectively these policies ensure coordination of maternal and infant HIV screening and care (if HIV-infected) with study evaluations.

10. Monitoring. The study will have annual monitoring of data entry and adherence to protocol performed by contract with a contract research organization with reports submitted to the sponsor, investigators, and IRBs.

11. Evaluations and laboratory studies

11.1 Dietary evaluation by 24 hour recall. Dietary evaluation will be based on multiple pass 24-hour recall. Kiswahili-speaking staff were trained in the use of this method during Study A. In addition the FANTA questionnaire will be administered at baseline, then every 3 months, to assess food insecurity.

11.2 Anthropometric measurements. Evaluation of body composition will include measurement of height, weight, 3-site skinfold thickness (triceps, subscapular and supailiac) and upper arm, waist and hip circumference. Fat free body mass and percent body fat will be calculated from anthropometric measurements using the equations of Durnin et al. Upper arm circumference and triceps skinfold measures will be used to calculate mid-arm muscle circumference or area, providing an estimate of body protein reserves.

11.3 Routine laboratory studies. CBC (complete blood count) and serum albumin levels will be determined at the MUHAS clinical laboratories using standard methods.

11.4 CD4 counts. Enumeration of CD4 numbers in HIV positive subject blood samples will be done on-site using anti-CD4 specific monoclonal antibodies and standardized flow cytometric methods. CD4 enumeration is routinely performed in the DarDar immunology laboratory in Dar es Salaam, and the laboratory is equipped with a FACScan flow cytometer (BD Biosciences Immunocytometry Systems) for this purpose.

11.5 Collection, preparation, storage and testing of breast milk. Maternal breast milk will be collected by either manual expression or by breast pump (Madela, McHenry, IL). From 3 to 10 mls of breast milk will be obtained from the right breast and placed in a sterile container. The containers will be coded on-site and samples immediately processed according to the requirements of the assays. Unprocessed samples may be stored at 4°C up to 4 hrs if staff is unable to process immediately.

After collection of whole milk, the volume of the milk will be measured and recorded. Two glass hematocrit tubes will immediately be filled with milk by capillary action and sealed at one end. For breast milk total cell count, 100 µl whole milk will be transferred to a tube in which 100 µl trypan blue has already been added. The remaining milk will be centrifuged at 400 x g for 15 minutes. The lipid and supernatant will be transferred together into a new container, aliquotted, and stored at – 80 °C in the DarDar laboratory until it is shipped on dry ice to Dartmouth. A drop of the remaining cell pellet will be extracted using a pipetteman, mixed 1:1 with 5% chicken ovalbumin in isotonic saline, and smeared onto a clean glass microscope slide for preparation of a differential cell count. The rest of the cell pellet will be frozen at -80° until shipped on dry ice to Dartmouth for further studies.

11.6 Breast milk cell count. 100 µl whole milk and 100 µ trypan blue will be mixed by pipetteman, and approximately 15 µl of the mixture will be added to a hemocytometer. Whole round, shiny cells (not cell particles or dead cells) will be counted as per whole blood. Results will be recorded in a laboratory notebook.

11.7. Breast milk differential cell count. The methods for preparing slides, for staining human milk using Wright's stain, and for differential cell counting will be performed as previously described [1 2 3]. In addition, five percent chicken ovalbumin (Sigma) in isotonic saline ensures adherence of the milk to the slide during the staining procedure. Briefly, 5% chicken ovalbumin solution will be mixed 1:1 with a small drop from the cell pellet , transferred to a clean glass slide, and smeared as per whole blood differential protocols. Slides will dry overnight in an air conditioned room, and will be heat fixed over an open flame prior to staining. Slides will be stained using Wright stain protocol (Protocol Wright's Stain, Protocol Wright Buffer, Fisher HealthCare, Houston, TX). Slides will air dry completely prior to immersing in xylene substitute, mounting, and coverslipping.

Cells will be counted per whole blood differential protocols, until a minimum of 200 cells have been counted. Cells will be differentiated into the categories of non-blood-derived cells (e.g.,mammary secretory epithelial cells, non-secretory epithelial cells), neutrophils, lymphocytes, monocytes/macrophages, eosinophils, and basophils. (Schalm, 1971, Chap 6; Max Paape , personal communication). If less than 200 cells are on the slide, all cells will be counted and differentiated. Resulted will be recorded in a laboratory notebook.

11.8 Breast milk immunology. Cytokines IL-6 and IL-8 will be measured using ELISA kits specifically designed for IL-8 and IL-6 (R&D Systems, Minneapolis, MN). Total breast milk IgA will be measured using a commercial assay.

11.9 Breast milk lipid concentration. Breast milk lipid concentration will be measured by the creamatocrit method. After collection of breast milk into sample containers, glass hematocrit tubes will immediately be filled with milk by capillary action and sealed at one end. Tubes will be spun in a hematocrit centrifuge for 15 minutes at 12,000 x g and the percentage lipid assessed by used of a hematocrit reader card [5].

11.10 Breast milk sodium and potassium. Milk Na and K will be measured by flame photometry, expressed as the Na/K ratio, and log transformed to obtain normal distributions for parametric analyses.

12. Data management. Standard case report forms (CRFs) were piloted in Study A and will be used for the Study. All data will be collected in Tanzania, double entered into a Filemaker database, justified and transferred electronically to the Data Center at Dartmouth without personal identifiers.

13. Community Advisory Board. A DarDar Community Advisory Board (CAB) in Dar es Salaam, Tanzania will be constituted and will meet every 6 months. The Board will consist of community and political leaders as well as men and women with HIV/AIDS and serves to advise Dartmouth and MUHAS study investigators on issues related to study ethics, recruitment and follow-up and helps to provide community support for the research project. Prior to inception the proposed nutritional study will be presented to the CAB for advice on study conduct, recruitment, and selection of the PCS. Updates will be provided for discussion at each scheduled CAB meeting during the study.

14. Contingencies. Based on our experience with previous US and international HIV studies we realize that there are numerous potential challenges to completing a research study on an HIV clinical trial as designed. The first of these relates policies for treatment of HIV, which may change during the years of the proposed study. For example, ART therapy might be recommended at an earlier stage of HIV disease or new

recommendations may be promulgated for nutritional supplementation. Randomization will address many potential confounders due to changing HIV policies. Our group works closely with HIV and TB thought leaders and policy makers in Tanzania and should have advance warning of changes in policy that might require a change in study design or patient management.

15. Significance. The inter-related study components of the present proposal will provide the first controlled data on the clinical effects of a PCS in HIV-infected pregnant and breastfeeding women vulnerable to malnutrition. Data on the effects of the PCS on critical health outcomes have the potential to influence international nutrition recommendations for women with HIV who are pregnant and subsequently breast feeding. In addition, the immune response data on breast milk has the potential to develop new hypotheses on the effects of PCS on breast milk transmission of HIV and to lead to nutritional interventions to help reduce maternal to infant transmission of HIV.

16. Timeline

Table 3. Timeline for Nutrition Study 2-B

Months	2	4	6	8	10	12	18	21	24	27	30
Recruitment											
Intervention and follow-up											
Final data analysis											

References

1. Ho FC, Wong RL, Lawton JW. Human colostral and breast milk cells. A light and electron microscopic study. Acta Paediatr Scand 1979;68:389-96.
2. Dulin A, Paape MJ, Wergin WP. Differentiation and enumeration of somatic cells in goat milk. J Food Prot 1982;45:435-9.
3. Schalm OW, Carroll EJ, Nain NC. Bovine mastitis Philadelphia: Lea and Febiger; 1971.
4. Ghosh MK, Kuhn L, West J, et al. Quantitation of human immunodeficiency virus type 1 in breast milk. J Clin Microbiol 2003;41:2465-70.
5. Lucas A, Gibbs JA, Lyster RL, JD B. Creamatocrit: simple clinical technique for estimating fat concentration and
energy value of human milk. . Br Med J 1978;22:1018-20.
