# Supplementary material for: Pre- and post-natal macronutrient supplementation for HIV–positive women in Tanzania: Effects on infant birth weight and HIV transmission
Source: PLoS One. 2018 Oct 11;13(10):e0201038. doi: 10.1371/journal.pone.0201038 (PMC6181269; doi:10.1371/journal.pone.0201038)
Supplement: S3 File — (ZIP) [file pone.0201038.s003.zip › dataset/Form AP 6-12-12.pdf]

Form AP
(anthropometry)

DarDar 2.0
~ Nutrition Study ~
version 2.0

Study ID #: ______________
Name - l: ______________
f: ______________

Anthropometry **See Appendix A for details)**

1. **Visit date** ____/____/______ (dd,MON,yyyy) **1a. Where?** 0 At IDC 1 At patient home
2. **Visit Site** 0 IDC 1 Home
3. **Height** ____________ m
4. **Weight** ____________ kg
5. **BMI** ____________ kg/m^2
6. **Circumferences**
   a. Mid-upper arm (use marked area) ____________ cm
   b. Waist (narrowest part of torso) ____________ cm
   c. Hip (maximum buttock extension) ____________ cm
   d. Thigh (use marked area) ____________ cm
7. **Skinfold thickness (use marked areas)**

   a. Triceps (back of mid-upper arm)
   Time 1 ____________ mm
   Time 2 ____________ mm
   Time 3 ____________ mm

   b. Subscapular (below the shoulder blade)
   Time 1 ____________ mm
   Time 2 ____________ mm
   Time 3 ____________ mm

   c. Suprailiac (above hip)
   Time 1 ____________ mm
   Time 2 ____________ mm
   Time 3 ____________ mm

   d. Abdomen
   Time 1 ____________ mm
   Time 2 ____________ mm
   Time 3 ____________ mm

   e. Thigh
   Time 1 ____________ mm
   Time 2 ____________ mm
   Time 3 ____________ mm

   Able to perform **Yes** ☐ 1 **No** ☐ 0 if no, reason: 0 Patient exceeded caliper limit
   1 Other ______________________

8. **Daily activity level**
   0 Very light (seated and standing activities such as church, riding the bus, waiting in clinic)
   1 Light (preparing meals, walking <30 minutes)
   2 Moderate (carrying a load, biking, walking ≥30 minutes)
   3 Heavy (heavy manual digging, walking with a load uphill)
9. **Comments:** ____
____
____
____
10. **Form completed by (study nurse):** ______________________
11. **Form checked by (MD or cons. dietician):** ______________________

Data entry 1 code: ________
Data entry 2 code: ________

© 2010, Trustees of Dartmouth College, Hanover, NH 03755 USA

Form updated:
1-Jul-11
