# Supplementary material for: Pre- and post-natal macronutrient supplementation for HIV–positive women in Tanzania: Effects on infant birth weight and HIV transmission
Source: PLoS One. 2018 Oct 11;13(10):e0201038. doi: 10.1371/journal.pone.0201038 (PMC6181269; doi:10.1371/journal.pone.0201038)
Supplement: S3 File — (ZIP) [file pone.0201038.s003.zip › dataset/Form B 6-12-12.pdf]

Form B
(baseline/enrollment)

Screening #: ____________

DarDar 2.0
~ Nutrition Study ~
version 2.0

Study ID #: ____________
Name - l: ____________
f: ____________

Baseline/Enrollment

1. **Visit date:** ____/____/______ (dd,MON,yyyy)
2. **Confirm subject name from screening form** — Yes ☐ 1 No ☐ 0
3. **Assign study ID #:** ____________ **Study A = 4000-4299; Study B = 4300-4599; Study C=4600-4899**
4. **Alternative names by which patient is known:**
   ____
   ____
5. **Current address:**
   Street/Area: ____________________
   ____
   City: Dar es Salaam, Tanzania
   Phone #: ____________________
6. **Length of time at current residence:** ______ yrs (If <1 yr, put 1)
7. **Place of employment (where relevant):** ____________________
8. **Address of employment/work:**
   Street/Area: ____________________
   ____
   City: ____________________
   Phone #: ____________________
9. **Name and address of 2 contacts (confidantes):**
   a. Name: ____________________
   Relationship: ____________________
   Street: ____________________
   ____
   City: ____________________
   Phone #: ____________________
   Place of employment: ____________________

   b. Name: ____________________
   Relationship: ____________________
   Street: ____________________
   ____
   City: ____________________
   Phone #: ____________________
   Place of employment: ____________________

10. **This page completed by (study nurse):** ____________________

Medical History

11. BCG scar and previous treatment for active tuberculosis?

a. **TB**
0 no
1 yes
if yes:
0 pulmonary
1 other ________

Date of treatment start: ___/___ (mm,yyyy)

Source of information (choose ONE)
0 history from pt
1 hospital or clinic record--> AFB smear | 0 neg 1 pos 9 unk |
2 history from family member/acquaintance

b. **Presence of BCG scar** 0 no
1 yes if yes, location: 1 right 2 left

12. Current treatment for active tuberculosis?

0 no
1 yes **if yes, date of treatment**
start: ___/___ (mm,yyyy) finish: ___/___ (mm,yyyy)

Source of information (choose ONE)
0 Green Card
1 Other ________

**drugs**	**Yes**	**No**
Unknown	☐1	
INH	☐1	☐0
Rifampin	☐1	☐0
PZA	☐1	☐0
Ethambutol	☐1	☐0
Streptomycin	☐1	☐0
Other ________	☐1	☐0

Source of information (choose ONE)
0 Green Card
1 Other ________

13. TB results from Green Card

date of smear	AFB smear
1. ___/___/___	☐0 neg ☐1 (1+) ☐2 (2+) ☐3 (3+) ☐4 pos, no quantity indicated

☐ Green Card not available: Explain ________

14. Current symptoms

	**Yes**	**No**	
nausea	☐1	☐0	
cough	☐1	☐0	(duration: ___wks) **(if <1 wk, enter days: ___)**
bloody cough (hemoptysis)	☐1	☐0	
weight loss (5 kg in last year)	☐1	☐0	☐ 9 BF, so not applicable
chronic diarrhea (>=3 stool/day for >=3 wks - now).	☐1	☐0	
fever	☐1	☐0	(duration: ___wks) **(if <1 wk, enter days: ___)**
excessive night sweats	☐1	☐0	
other	☐1	☐0	

specify: ________________________________

15. Other prior opportunistic infection?

	**Yes**	**No**	**date (mm,yyyy)**
herpes zoster (shingles)	☐1	☐0	___/___
pneumonia	☐1	☐0	___/___
thrush	☐1	☐0	___/___
chronic diarrhea (>=3 stool/day for >=3 wks - past).	☐1	☐0	___/___
Kaposi's sarcoma	☐1	☐0	___/___

© 2010, Trustees of Dartmouth College, Hanover, NH 03755 USA

Form B
(baseline/enrollment)

DarDar 2.0 ~ Nutrition Study ~
version 2.0

Study ID #: ____________
Name - l: ____________
f: ____________

Screening #: ____________

16. Medications today

	Yes	No
HIV antiviral therapy	☐ 1	☐ 0
trimethoprim/sulfa	☐ 1	☐ 0
malaria treatment	☐ 1	☐ 0
iron therapy	☐ 1	☐ 0
Folic acid	☐ 1	☐ 0
isoniazid (INH) for latent TB (pos PPD)	☐ 1	☐ 0
traditional medicines, specify: ____________	☐ 1	☐ 0
other vitamins, minerals, specify: ____________	☐ 1	☐ 0
other, specify: ____________	☐ 1	☐ 0

→ **If yes, drugs:**

	Yes	No	date started: (mm,yyyy)
AZT	☐ 1	☐ 0	/
3TC	☐ 1	☐ 0	/
NVP	☐ 1	☐ 0	/
EFV	☐ 1	☐ 0	/
Ritonavir/Lopinavir	☐ 1	☐ 0	/
Other protease inhibitors: ____________	☐ 1	☐ 0	/
Other ART ____________	☐ 1	☐ 0	/

For TB patients (Study C only)

	Yes	No
**17. Sputum #1 obtained today?**	☐ 1	☐ 0
**18. 2 or 3 sputum cups given to patient?**	☐ 1	☐ 0
**19. Other lab?** ____________	☐ 1	☐ 0
**20. Chest x-ray obtained?**	☐ 1	☐ 0

If unable to produce expectorated sputum today, request sputum induction
Instruct patient to collect 2 additional sputa each of next 2 mornings, keep as cool as possible, and bring back in 2 days.

For BF patients (Study B only)

	Yes	No
**21. Ultasound obtained?**	☐ 1	☐ 0

Give print out to patient

For all patients

Go to Form R for other lab studies

	Yes	No
**22. Nutrition education given?**	☐ 1	☐ 0

23. Open envelope to determine randomization and record:

1 MNS **Give 1 month supply, and explain how to take**
2 MNS & PCS **Give 2 week supply, and explain how to take**

a. if 2, current number of children age <= 7 in same household ____________
b. if age >=1, then give 4 PCS packets per child every 2 weeks - number of packets: ____________

	Yes	No
**24. Other lab?** ____________	☐ 1	☐ 0
**25. Study photo ID taken?** (B & C only)	☐ 1	☐ 0

26. Form completed by: ____________

27. Plant Tuberculin skin tests: (Study C only)
Administer 0.1 ml PPD (2 TU RT-23Copenhagen) intradermally on left forearm Yes ☐ 1 No ☐ 0

28. Skin tests by (technical nurse): ____________ **time:** ____________

29. Date of next scheduled visit: ____/____/____ (dd,MON,yyyy)
return in 2 or 3 days to read TST, and routine visit in 1 month

30. Form checked by (study MD): ____________

31. Comments: ____________

Proceed to Forms AP, DEI and FI for all, Form T for Study C

Data entry 1 code: ________
Data entry 2 code: ________

© 2010, Trustees of Dartmouth College, Hanover, NH 03755 USA
