# Supplementary material for: Pre- and post-natal macronutrient supplementation for HIV–positive women in Tanzania: Effects on infant birth weight and HIV transmission
Source: PLoS One. 2018 Oct 11;13(10):e0201038. doi: 10.1371/journal.pone.0201038 (PMC6181269; doi:10.1371/journal.pone.0201038)
Supplement: S3 File — (ZIP) [file pone.0201038.s003.zip › dataset/Form DE 7-26-12.pdf]

Form DE
24 Hour Dietary Evaluation

DarDar 2.0
~ Nutrition Study ~
version 2.0

Study ID #: ____________
Name - l: ____________
f: ____________

INSTRUCTIONS:

This section should be filled out by the interviewer using the multiple pass method. Ask the participant to list aloud for you all food and beverages that she consumed during the previous day from the time she woke up until the time she went to bed, including the time that each food or beverage was consumed. All foods and beverages, including alcoholic beverages, vitamin supplements, and all non-prescription medications are recorded on the recall form.
If fasting yesterday, reschedule.

Time (24 hr)	1=Meal 2=Snack 3=Beverage	Food/Beverage description	Ingredients	Type	Measure Utensil	Frac-tion	Weight (g)	Food Code
ex. 1330	*1*	*Dagaa relish with oil*	*dagaa, tomato, onion, salt, oil*	*9*	*4*	*0.5*	*50*	*557*

Utensil code: 1 =bowl, 2 =glass, 3 =cup, 4 =plate, 5 =tablespoon, 6 =teaspoon, 7 =saucer, 8 =serving (see Apx C2)
Type code: 1, 2, 3, 4, or 9 (see Tanzanian Nutrient Food tables)

Form DE
24 Hour Dietary Evaluation

DarDar 2.0
~ Nutrition Study ~
version 2.0

Study ID #: ____________
Name - l: ____________
f: ____________

Date of interview ____/____/________ (dd,MON,yyyy) **Category** ☐TB/HIV ☐BF/HIV
Time of interview ____________ **Where?** 0 At IDC 1 At patient home
Number of DE 1 DE1 2 DE2 3 DE3 and above

Ask the participant to answer the following questions. Read the responses aloud to her.

1. **How would you describe the amount of food you ate yesterday?**
   0 considerably less 1 typical 2 considerably more
   a. **If not typical, why?** 1 illness 2 travel 3 food not available 4 other ____________
2. **When was the last time you ATE anything prior to your visit today?**
   0☐ <5 hrs before visit 1☐ 5-8 hrs before visit 2☐ > 8 hours before visit
3. **When was the last time you DRANK anything prior to your visit today?**
   0☐ <5 hrs before visit 1☐ 5-8 hrs before visit 2☐ > 8 hours before visit

---

4. **Interviewer's opinion of information**
   0 unreliable 1 reliable
   a. **If unreliable, why?** 0 unable to recall 1 or more meals 1 other ____________

---

5. **Number of other people living in the household in the past 4 weeks** ______ **Ages:** ____________
6. **Are you taking ART medications?** ☐1 ☐0
   a. **If yes, drugs:**

	Yes	No	date started: (mm,yyyy)
AZT.	☐1	☐0	___/______
3TC	☐1	☐0	___/______
NVP.	☐1	☐0	___/______
EFV.	☐1	☐0	___/______
Ritonavir/Lopinavir. . . .	☐1	☐0	___/______
Other protease inhibitors: ____________	☐1	☐0	___/______
Other ART. . . . . ____________	☐1	☐0	___/______

   b. **In the past 4 weeks, did you ever NOT take your HIV medication?** Yes ☐1 No ☐0
      1. **If yes, how many days did you miss one or more doses?** ____________
      2. **If yes, for what reason(s)?**

	Yes	No
Ran out of medication.	☐1	☐0
Forgot.	☐1	☐0
Afraid of side effects.	☐1	☐0
Feeling better.	☐1	☐0
HIV meds made me too hungry.	☐1	☐0
Did not have enough food.	☐1	☐0
Other, specify ____________	☐1	☐0

7. **Do you use vitamins, herbs, traditional medicines?** ☐1 ☐0 **[if yes, instruct pt to bring for next visit]**
8. **Are you still exclusively breastfeeding?** ☐1 ☐0 **Explain:** ____________
9. **Date of next scheduled visit:** ____/____/______ (dd,MON,yyyy)
10. **Appointment card completed?** ☐1 ☐0
11. **Patient paid?** ☐1 ☐0
12. **Form completed by:** ____________
13. **Form checked by (MD or cons. dietician):** ____________
14. **Comments:**

Data entry 1 code: ________
Data entry 2 code: ________

© 2010, Trustees of Dartmouth College, Hanover, NH 03755 USA
