# Supplementary material for: Pre- and post-natal macronutrient supplementation for HIV–positive women in Tanzania: Effects on infant birth weight and HIV transmission
Source: PLoS One. 2018 Oct 11;13(10):e0201038. doi: 10.1371/journal.pone.0201038 (PMC6181269; doi:10.1371/journal.pone.0201038)
Supplement: S3 File — (ZIP) [file pone.0201038.s003.zip › dataset/Form F 9-19-12.pdf]

# DarDar 2.0
# ~ Nutrition Study ~

version 2.0

**Study ID #:** ____________

Name - l: ____________

f: ____________

## Form F (2B)

1. **Visit date** ____/____/________ (dd,MON,yyyy) **1a. Where?** 0 At IDC 1 At patient home
2. **Consumption of MNS (all subjects)**
   a. How many days in the past month did you take MNS? ________ days
3. **Consumption of PCS (PCS subjects only)**
   a. How many days in the past month did you take **any** PCS? ________ days
   b. How many days in the past month did you take **all** PCS? ________ days
   c. Estimate how much of PCS you normally took each day? ________ %
   d. How many times did you usually take the PCS each day? ________
   e. Did you normally take the PCS with other food or by itself? 0 with other food 1 PCS alone
   f. How often did you share your PCS in the past month? 0 never; 1 sometimes: ________ days; 2 every day
   g. How does the PCS taste now? 1 Bad 2 Somewhat bad 3 Somewhat good 4 Very good
4. **Breastfeeding (all subjects in Study 2B)**
   a. Are you still breastfeeding every day? Yes ☐1 No ☐0 How many times per day? ________
   b. Have other foods or liquids been given to your baby in the past week?

| | Yes | No | |
|---|---|---|---|
| water. . . . . . . . . . . . . . . . . . . . . . . . . . . . . . | ☐1 | ☐0 | if yes, number of times per day: ________ |
| other 1, specify ________ | ☐1 | ☐0 | if yes, number of times per day: ________ |
| other 2, specify ________ | ☐1 | ☐0 | if yes, number of times per day: ________ |

5. **Mother's health (since last visit)**

**a. Current Medications**

| | Yes | No |
|---|---|---|
| HIV antiviral therapy. . . . . . . . . . . . . . . . . . . . . . . . . . . . | ☐1 | ☐0 |
| trimethoprim/sulfa. . . . . . . . . . . . . . . . . . . . . . . . . . . | ☐1 | ☐0 |
| malaria treatment. . . . . . . . . . . . . . . . . . . . . . . . . | ☐1 | ☐0 |
| iron therapy. . . . . . . . . . . . . . . . . . . . . . . . . . . . . . . . . | ☐1 | ☐0 |
| folic acid. . . . . . . . . . . . . . . . . . . . . . . . . . . . . . . . . . | ☐1 | ☐0 |
| isoniazid (INH) for latent TB (pos PPD). . . . . . . . . | ☐1 | ☐0 |
| traditional medicines, specify: ________ | ☐1 | ☐0 |
| vitamins, minerals, specify: ________ | ☐1 | ☐0 |
| other, specify: ________ | ☐1 | ☐0 |

→ **If yes, drugs:**

| | Yes | No | **date started:** (mm,yyyy) |
|---|---|---|---|
| AZT. . . . . . . . . . . . . . . . . | ☐1 | ☐0 | ____/______ |
| 3TC . . . . . . . . . . . . . . . . | ☐1 | ☐0 | ____/______ |
| NVP. . . . . . . . . . . . . . . . . | ☐1 | ☐0 | ____/______ |
| EFV. . . . . . . . . . . . . . . . . | ☐1 | ☐0 | ____/______ |
| Ritonavir/Lopinavir. . . . | ☐1 | ☐0 | ____/______ |
| Other protease inhibitors: ________ | ☐1 | ☐0 | ____/______ |
| Other ART. . . . . . . . . . . ________ | ☐1 | ☐0 | ____/______ |

**b. Illness**

| | Yes | No | |
|---|---|---|---|
| Mastitis. . . . . . . . . . . . . . . . . . . . . . | ☐1 | ☐0 | if yes, describe: ________ |
| Diarrhea (>= 3 loose stools/day) . . . | ☐1 | ☐0 | if yes, describe: ________ |
| Respiratory infection. . . . . . . . . . . | ☐1 | ☐0 | if yes, describe: ________ |
| Skin infection . . . . . . . . . . . . . . . . | ☐1 | ☐0 | if yes, describe: ________ |
| Other, specify: ________ | ☐1 | ☐0 | if yes, describe: ________ |

# DarDar 2.0
# ~ Nutrition Study ~

version 2.0

**Study ID #:** ____________

Name - l: ____________

f: ____________

**INFANT**

**Complete questions 6-9 once, on first visit after delivery**

6. **Birth date** ___/___/______ (dd,MON,yyyy)
7. **Birth outcome** 0 Stillborn 1 Live birth
8. **Sex** 0 Male 1 Female
9. **Birth weight** ____________ kg

**Complete questions 10-16 for each infant visit**

10. **Weight today** ____________ kg
11. **Height today** ____________ cm
12. **Temperature** ____________ C
13. **Status** 0 healthy
    1 current illness — **Have DPP evaluation and complete Q16**
    2 deceased

    Date of death ___/___/______ (dd,MON,yyyy)

    Cause of death: ____________

**14. Illness**

| | Yes | No | |
|---|---|---|---|
| Cord sepsis. . . . . . . . . . . . . . . . . . . | ☐ 1 | ☐ 0 | |
| Diarrhea (>= loose stools/day) . . . . | ☐ 1 | ☐ 0 | if yes, describe: ____________ |
| Respiratory infection. . . . . . . . . . . | ☐ 1 | ☐ 0 | if yes, describe: ____________ |
| Skin infection . . . . . . . . . . . . . . . . | ☐ 1 | ☐ 0 | if yes, describe: ____________ |
| Otitis media. . . . . . . . . . . . . . . . . . | ☐ 1 | ☐ 0 | if yes, describe: ____________ |
| Other, specify: ____________ | ☐ 1 | ☐ 0 | if yes, describe: ____________ |

**15. Current Medications**

| | Yes | No |
|---|---|---|
| HIV antiviral therapy. . . . . . . . . . . . . . . . . . . . . . . . . . . | ☐ 1 | ☐ 0 |
| trimethoprim/sulfa. . . . . . . . . . . . . . . . . . . . . . . . . | ☐ 1 | ☐ 0 |
| malaria treatment. . . . . . . . . . . . . . . . . . . . . . . . | ☐ 1 | ☐ 0 |
| iron therapy. . . . . . . . . . . . . . . . . . . . . . . . . . . . . . . . | ☐ 1 | ☐ 0 |
| folic acid. . . . . . . . . . . . . . . . . . . . . . . . . . . . . . . . | ☐ 1 | ☐ 0 |
| isoniazid (INH) for latent TB (pos PPD). . . . . . . . . | ☐ 1 | ☐ 0 |
| traditional medicines, specify: ____________ | ☐ 1 | ☐ 0 |
| vitamins, minerals, specify: ____________ | ☐ 1 | ☐ 0 |
| other, specify: ____________ | ☐ 1 | ☐ 0 |

→ **If yes, drugs:**

| | Yes | No | (mm,yyyy) |
|---|---|---|---|
| AZT. . . . . . . . . . . . . . . . | ☐ 1 | ☐ 0 | ___/___ |
| 3TC . . . . . . . . . . . . . . . | ☐ 1 | ☐ 0 | ___/___ |
| NVP. . . . . . . . . . . . . . . . | ☐ 1 | ☐ 0 | ___/___ |
| EFV. . . . . . . . . . . . . . . . | ☐ 1 | ☐ 0 | ___/___ |
| Ritonavir/Lopinavir. . . . | ☐ 1 | ☐ 0 | ___/___ |
| Other protease inhibitors: ____________ | ☐ 1 | ☐ 0 | ___/___ |
| Other ART. . . . . . . . . . . ____________ | ☐ 1 | ☐ 0 | ___/___ |

16. **Examination and comments by MD:** ____________

17. **Form completed by (study nurse):** ____________
18. **Form checked by (cons. dietician):** ____________

**Data entry 1 code:** ______
**Data entry 2 code:** ______

© 2010, Trustees of Dartmouth College, Hanover, NH 03755 USA

Form updated: 1-Jul-2011
