# Supplementary material for: Pre- and post-natal macronutrient supplementation for HIV–positive women in Tanzania: Effects on infant birth weight and HIV transmission
Source: PLoS One. 2018 Oct 11;13(10):e0201038. doi: 10.1371/journal.pone.0201038 (PMC6181269; doi:10.1371/journal.pone.0201038)
Supplement: S3 File — (ZIP) [file pone.0201038.s003.zip › dataset/Form FI 9-19-12.pdf]

Form FI
(food insecurity)

DarDar 2.0
~ Nutrition Study ~

version 2.0

Study ID #: ____________
Name - l: ____________
f: ____________

Household Food Insecurity Access Scale (HFIAS)

1. Visit date ____/____/________ (dd,MON,yyyy) **1a. Where?** 0 At IDC 1 At patient home

**In the past 4 weeks:**	**Yes**	**No**		**Rarely (1-2 x in past 4 wks)**	**Sometimes (3-10 x in past 4 wks)**	**Often (>10 x in past 4 wks)**
**2. Did you worry that your household would not have enough food?**	☐1	☐ 0	**If yes, how often**	☐0	☐ 1	☐ 2
**3. Were you or any household member not able to eat the kinds of food you preferred because of a lack of resources?**	☐1	☐ 0	**If yes, how often**	☐0	☐ 1	☐ 2
**4. Did you or any household member have to eat a limited variety of foods because of a lack of resources?**	☐1	☐ 0	**If yes, how often**	☐0	☐ 1	☐ 2
**5. Did you or any household member have to eat some foods that you really did not want to eat because of a lack of resources**	☐1	☐ 0	**If yes, how often**	☐0	☐ 1	☐ 2
**6. Did you or any household member have to eat a smaller meal than you felt you needed because there was not enough food?**	☐1	☐ 0	**If yes, how often**	☐0	☐ 1	☐ 2
**7. Did you or any household member have to eat fewer meals in a day because there was not enough food?**	☐1	☐ 0	**If yes, how often**	☐0	☐ 1	☐ 2
**8. Was there ever no food to eat of any kind in your household because of lack of resources?**	☐1	☐ 0	**If yes, how often**	☐0	☐ 1	☐ 2
**9. Did you or any household member go to sleep at night hungry because there was not enough food?**	☐1	☐ 0	**If yes, how often**	☐0	☐ 1	☐ 2
**10. Did you or any household member go a whole day and night without eating anything because there was not enough food?**	☐1	☐ 0	**If yes, how often**	☐0	☐ 1	☐ 2

DarDar 2.0
~ Nutrition Study ~

version 2.0

Study ID #: ____________
Name - l: ____________
f: ____________

11. What is your main staple food?
0 Maize
1 Rice
2 Bananas
3 Sorghum/Millet
4 Cassava
5 Other ____________

---

12. Where do you get the staple food from?
0 Shop/Market
1 Farm
2 Food aid/gift
3 Other ____________

---

13. If you don't have enough food, how is it divided in the household?
0 Equally to all
1 First to men
2 First to children
3 First to me because of condition
4 Lives alone
5 Other ____________

How often do you eat:

	Never	Occasionally	Weekly	Daily	Source: Shop/ Market	Farm	Food aid/ Gift	Other	
**14. Nuts.**	☐ 0	☐ 1	☐ 2	☐ 3	☐ 0	☐ 1	☐ 2	☐ 3	______
**15. Beans.**	☐ 0	☐ 1	☐ 2	☐ 3	☐ 0	☐ 1	☐ 2	☐ 3	______
**16. Fish.**	☐ 0	☐ 1	☐ 2	☐ 3	☐ 0	☐ 1	☐ 2	☐ 3	______
**17. Milk.**	☐ 0	☐ 1	☐ 2	☐ 3	☐ 0	☐ 1	☐ 2	☐ 3	______
**18. Eggs.**	☐ 0	☐ 1	☐ 2	☐ 3	☐ 0	☐ 1	☐ 2	☐ 3	______
**19. Chicken.**	☐ 0	☐ 1	☐ 2	☐ 3	☐ 0	☐ 1	☐ 2	☐ 3	______
**20. Meat.**	☐ 0	☐ 1	☐ 2	☐ 3	☐ 0	☐ 1	☐ 2	☐ 3	______
**21. Fruits.**	☐ 0	☐ 1	☐ 2	☐ 3	☐ 0	☐ 1	☐ 2	☐ 3	______
**22. Vegetables.**	☐ 0	☐ 1	☐ 2	☐ 3	☐ 0	☐ 1	☐ 2	☐ 3	______
**23. Grain (rice, maize,).**	☐ 0	☐ 1	☐ 2	☐ 3	☐ 0	☐ 1	☐ 2	☐ 3	______

24. Comments: ____
____
____

25. Form completed by (study nurse): ____________________

26. Form checked by (MD or cons. dietician): ____________________

Proceed to 24 Hour Dietary Evaluation form (DE)

Data entry 1 code: ________
Data entry 2 code: ________

© 2010, Trustees of Dartmouth College, Hanover, NH 03755 USA
