# Supplementary material for: Pre- and post-natal macronutrient supplementation for HIV–positive women in Tanzania: Effects on infant birth weight and HIV transmission
Source: PLoS One. 2018 Oct 11;13(10):e0201038. doi: 10.1371/journal.pone.0201038 (PMC6181269; doi:10.1371/journal.pone.0201038)
Supplement: S3 File — (ZIP) [file pone.0201038.s003.zip › dataset/Form R 9-19-12.pdf]

Form R
(results of lab studies)

☐ Subject (mother)
☐ Infant

DarDar 2.0
~ Nutrition Study ~

version 2.0

Study ID #: ____________
Name - l: ____________
f: ____________

Results of lab studies

Note: Keep Form R in 'pending' bin until all 3 AFB smears and CXR results for possible TB are reported.

1. **Form date:** ___/___/______ (dd,MON,yyyy)
2. **Related to form?** ________ **from visit date?** ___/___/______ (dd,MON,yyyy)

3. **Sputum results:**

date obtained	Neg	Pos	AFB smear	Neg	Pos	culture
1. ___/___/___ ☐1 expectorated ☐2 induced	☐0	☐1	☐0 1-9 (# organisms) ____ ☐1 (1+) ☐2 (2+) ☐3 (3+) ☐4 conc. date results recorded ___/___/___	☐0	☐1	☐0 1-9 (# organisms) ____ ☐1 (1+) ☐2 (2+) ☐3 (3+) ☐4 contam ☐5 non-tuber. myco date results recorded ___/___/___
2. ___/___/___ ☐1 expectorated ☐2 induced	☐0	☐1	☐0 1-9 (# organisms) ____ ☐1 (1+) ☐2 (2+) ☐3 (3+) ☐4 conc. date results recorded ___/___/___	☐0	☐1	☐0 1-9 (# organisms) ____ ☐1 (1+) ☐2 (2+) ☐3 (3+) ☐4 contam ☐5 non-tuber. myco date results recorded ___/___/___
3. ___/___/___ ☐1 expectorated ☐2 induced	☐0	☐1	☐0 1-9 (# organisms) ____ ☐1 (1+) ☐2 (2+) ☐3 (3+) ☐4 conc. date results recorded ___/___/___	☐0	☐1	☐0 1-9 (# organisms) ____ ☐1 (1+) ☐2 (2+) ☐3 (3+) ☐4 contam ☐5 non-tuber. myco date results recorded ___/___/___

4. **Chest x-ray:**

date done: ___/___/______ (dd,MON,yyyy)

read by: ____________
name

Result	Yes	No
infiltrates.	☐1	☐0
adenopathy.	☐1	☐0
if yes, calcified?----->	☐1	☐0
scarring.	☐1	☐0
pleural thickening	☐1	☐0
pleural effusion	☐1	☐0
granulomata	☐1	☐0
if yes, calcified?----->	☐1	☐0
cavities	☐1	☐0
miliary	☐1	☐0

Conclusion
0 not suspicious for TB
1 consistent with prior TB
2 consistent with active TB
3 other: ____________
____

date results recorded (dd,MON,yyyy)
___/___/______

notes:
....
....
....
....
....
....

5. **Other lab results:**

**date obtained** (dd,MON,yyyy)		**results**	**date results recorded** (dd,MON,yyyy)
___/___/___	a. CD4	________	___/___/___
___/___/___	b. viral load	________	___/___/___
___/___/___	c. Hct	________	___/___/___
___/___/___	d. Hb	________	___/___/___
___/___/___	e. WBC	________	___/___/___
___/___/___	f. albumin	________	___/___/___
___/___/___	g. HIV elisa	________	___/___/___
___/___/___	h. other	________	___/___/___
___/___/___	i. other	________	___/___/___

6. **Tuberculin skin test result** ______ mm **date read:** ___/___/______ (dd,MON,yyyy)

7. **Ultrasound result**

a. BPD ____________ cm
b. HC ____________ cm
c. AC ____________ cm
d. FL ____________ cm
e. EFW ____________ kg

Form R
(results of lab studies)

☐ Subject (mother)
☐ Infant

DarDar 2.0
~ Nutrition Study ~

version 2.0

Study ID #: ______

Name - l: ______

f: ______

For mother only

	Yes	No
**8. Breast milk sample obtained today?**	☐ 1	☐ 0

a. Which breast? 0 Right 1 Left

b. Last feeding or milking from this breast 0 < 1 hour
1 >1 hour: how many ____hrs

**9. Problems with your breasts?**	Yes	No
cracked or sore nipples.	☐ 1	☐ 0
painful, swollen, or reddened breasts . .	☐ 1	☐ 0
bleeding nipples.	☐ 1	☐ 0
discharge other than milk from breasts	☐ 1	☐ 0
other	☐ 1	☐ 0

10. Laboratory studies today

	Yes	No
CBC (3mL purple top tube).	☐ 1	☐ 0
Albumin (3mL green top tube).	☐ 1	☐ 0
CD4 count (3mL purple top tube).	☐ 1	☐ 0
HIV viral load (7mL PPT tube).	☐ 1	☐ 0
Breast milk sample.	☐ 1	☐ 0
Other ______	☐ 1	☐ 0
Other ______	☐ 1	☐ 0

Infant

	Yes	No
HIV viral load (PPT tube).	☐ 1	☐ 0
HIV ELISA (3mL green top tube).	☐ 1	☐ 0
Other ______	☐ 1	☐ 0

11. Form completed by (clinical officer): ______

12. Form checked by (study MD): ______

13. Comments: ______

______

______

Data entry 1 code: ______
Data entry 2 code: ______

© 2010, Trustees of Dartmouth College, Hanover, NH 03755 USA

Form updated:
1-Dec-2010

Form R
(results of lab studies)

☐ Subject (mother)
☐ Infant

DarDar 2.0
~ Nutrition Study ~
version 2.0

Study ID #: ____________

Name - l: ____________

f: ____________
