# Supplementary material for: Pre- and post-natal macronutrient supplementation for HIV–positive women in Tanzania: Effects on infant birth weight and HIV transmission
Source: PLoS One. 2018 Oct 11;13(10):e0201038. doi: 10.1371/journal.pone.0201038 (PMC6181269; doi:10.1371/journal.pone.0201038)
Supplement: S3 File — (ZIP) [file pone.0201038.s003.zip › dataset/Form S 6-12-12.pdf]

Form S
(screening)

DarDar 2.0
~ Nutrition Study ~
version 2.0

Study ID #: ______________
Name - l: ______________
f: ______________

Screening #: ____________

1. **Visit date:** ____/____/______ (dd,MON,yyyy)

	Yes	No	
2. **Has study been described to patient?**	☐ 1	☐ 0	
3. **Has study consent form been signed?**	☐ 1	☐ 0	[Required for study participation]

4. **Date of birth:** ____/____/______ (dd,MON,yyyy) **If unknown, ask patient to estimate age** ______

5. **Place of birth:** 0 Tanzania 1 Other country: ______________

6. **Race:** 0 Tanzanian, African origin 2 Tanzanian, Arab origin
1 Tanzanian, Indian origin 3 Other: ______________

7. **Marital status:** 0 Single 2 Divorced 4 Cohabitating
1 Married 3 Widowed

8. **Highest level of education:** 0 None 2 Primary 4 Secondary
1 Some primary 3 Some Secondary 5 Higher

9. **Patient occupation:** ______________

10. **Partner occupation:** ______________

11. **How much do you spend each day on food for the family in your household?** ______________ (TSh)

12. **Number of other people living in household in the last month:** ______ **Ages:** ______________

Eligibility:

		Yes	No	
13.	age >=18?.	☐ 1	☐ 0	
14.	Residence in Dar?.	☐ 1	☐ 0	[If No, patient is NOT eligible. Go to Q#23]
15.	Female?.	☐ 1	☐ 0	
16.	Current Rx for opportunistic infection?	☐ 1	☐ 0	infection: ______________
17.	Currently in another HIV or TB study?	☐ 1	☐ 0	[If Yes, patient is NOT eligible. Go to Q#23]

PREGNANT PATIENTS

18. Planning exclusive breastfeeding? ☐ 1 ☐ 0

NOTE: If no HIV result in chart, BF patient is NOT eligible, go to Q#23

a. HIV result by clinic record or by report from clinic nurse? 0 Neg 1 Pos 2 Unk
if pos, CD4: __________ /mm³ date: ____/____/______ (dd,MON,yyyy)
☐ no CD4 available

b. Estimated date of delivery ____/____/______ (dd,MON,yyyy) If < 3 months away, patient is NOT eligible

c. High risk pregnancy? Yes ☐ 1 No ☐ 0 if yes, describe: 1 diabetes
2 pre-eclampsia
3 other ______________

[If Yes, patient is NOT eligible. Go to Q#23]

TB PATIENTS

19. AFB smear positive? ☐ 1 ☐ 0 [If No, patient is NOT eligible. Go to Q#23]

a. On TB Tx? ☐ 1 ☐ 0 Date Tx started: ____/____/______ (dd,MON,yyyy) [If >2 wks ago, patient is NOT eligible. Go to Q#23]

b. NTLP HIV result 0 Neg 1 Pos [Only eligible if result done]

HIV testing:

	Yes	No	
**20. Pre-test HIV counseling completed?**	☐ 1	☐ 0	
**21. Blood collected:**			
**a. 1 tiger top SST 4 ml (rapid HIV)**	☐ 1	☐ 0	Note: Have patient wait for HIV result
	**Pos**	**Neg**	[If both POS, mark yes for HIV pos on Q#22]
Rapid HIV #1 (Bioline)	☐ 1	☐ 0	[If both NEG, mark no for HIV pos on Q#22]
Rapid HIV #2 (Determine)	☐ 1	☐ 0	[If one POS and one NEG, mark no for HIV pos on Q#22]
	**Yes**	**No**	
**b. 1 lavender top 4 ml (CD4 count)**	☐ 1	☐ 0	

Eligibility: **Yes** **No**

22. HIV pos by DarDar? ☐ 1 ☐ 0

if yes or indeterminate, is patient in care at a CTC? **Yes** ☐ 1 **No** ☐ 0 — Refer to CTC if not already in care at CTC

CTC now or where referred to: ____________

if yes, current ART:
- 0 None, does NOT meet criteria for long term ART
- 1 None, but does meet criteria for long term ART, indication____________
- 2 Short term nevirapine for PMTCT only
- 3 Short term other for PMTCT only
  drugs:____________
- 4 Long term tx with 3 or more drugs

23. Category:
- 0 Ineligible
- 1 TB/HIV [TB eligible, HIV pos eligible, not breastfeeding]
- 2 BF/HIV [BF eligible, HIV pos eligible, not on TB Tx]

If 0, go to Q#26

If 1 or 2, return in 2 days for CD4 results, then proceed to Form B at next visit

24. Date/time of next scheduled visit: 0 morning ____/____/____ (dd,MON,yyyy)

25. Appointment card completed. . . . . ☐ 1 ☐ 0

26. Patient paid. . . . . ☐ 1 ☐ 0

27. Comments: ____________

____

____

28. Form completed by (study nurse): ____________

29. Form checked by (study MD): ____________

© 2010, Trustees of Dartmouth College, Hanover, NH 03755 USA
